# Job-Seeking Anxiety and Job Preparation Behavior of Undergraduate Students

**DOI:** 10.3390/healthcare10020288

**Published:** 2022-02-01

**Authors:** Jeoungmi Kim, Jina Oh, Vasuki Rajaguru

**Affiliations:** 1 Department of Nursing Science, Kaya University, Gimhae 50830, Korea; jeoung66@kaya.ac.kr; 2 College of Nursing & Institute of Health Science, Inje University, Busan 47392, Korea; ohjina@inje.ac.kr; 3 Department of Healthcare Management, Graduate School of Public Health, Yonsei University, Seoul 03722, Korea

**Keywords:** job anxiety, job preparation, behavior, university students, job search

## Abstract

This study attempted to examine and compare the job-seeking anxiety and job preparation behavior of undergraduate students. A descriptive cross-sectional study was employed; the study participants were 360 students (3rd and 4th grade), selected from K’ university in G city. Data were collected by structured self-reported questionnaires from November 2020 to February 2021. Variables included general characteristics, job-seeking anxiety and job preparation behavior and were analyzed by descriptive statistics, Pearson’s correlation, and multiple regression analysis by using the SPSS/WIN 25.0 program. Of the total population, 70.8% were female in the health and social science group, the age group was 22–24 years (55.2%; 50.2%) and were fourth grade 62%; 59.1%). The level of job-seeking anxiety of students showed a higher proportion in health science (4.45 ± 0.81) than social science (3.73 ± 0.55). The level of job preparation behavior also revealed the same results in health science (4.28 ± 0.76) and social science (4.06 ± 0.81). Job anxiety showed a positive correlation with employment anxiety induction situation (r = 0.32, *p* < 0.01) and employment anxiety induction causes (r = 0.27, *p* < 0.01), and social science students showed a positive correlation with employment anxiety induction situation (r = 0.24, *p* < 0.01) and employment anxiety induction causes (r = 0.23, *p* < 0.01). The factors of age, gender and desired job position are highly associated with job-seeking anxiety and job preparation behavior. The findings of this study revealed job-seeking anxiety was higher among the undergraduate students and showed a high level of job preparation behavior. There is a need to develop intervention strategies for promoting job preparation behavior and reducing job-seeking anxiety among undergraduate students by providing career planning to improve the positive attitude towards desired job selection.

## 1. Introduction

As the job crisis continues due to the economic recession, the problem of college students acquiring a job is becoming a significant issue in our society. According to statistics released in 2015 regarding annual employment trend published by the National Statistical Office, the youth unemployment rate was 9.2, up 0.2% from the previous year [1,2]. However, the actual unemployment rate is significantly higher than the official unemployment rate of the National Statistical Office [2]. According to the Ministry of Science, ICT and Future Planning’s report on the design and research of indexes for youth employment, the unemployment rate for youth in 2017 was estimated to be 34%, if hidden unemployed people that are not incorporated in statistics were included in the unemployment rate statistics [2,3].

The unemployment of youths in their twenties leads to weaker mental health status, including stress, anxiety, self-identity, and lack of job preparation tendencies compared to the employed group [4,5,6]. Previous research has shown that there is a positive relationship between youth unemployment and deterioration of mental health [7,8,9,10]. As a result of a longitudinal study for this purpose, unemployment was found to be correlated with neurotic complaints, such as depression, insomnia, poor concentration, and anxiety [10].

In addition, since the career process and job performance of college students depend on individual characteristics, the socioeconomic background of the family and school characteristics, they are placed in a social atmosphere in which they must choose a college with a good reputation for an entrance examination [11,12]. A study reported that undergraduate students, who have completed formal education, believe that their success can be identified through their job [8,13,14,15]. However, it is difficult for young people to find a job, which leads to an increasing unemployment status of undergraduate students as soon as they have completed their course [15,16]. In fact, the university counseling center and the student life research institute have shown that their anxiety and stress regarding work are quite high [15]. For college students, acquiring a job is a major issue in relation to economic independence; however, it can pose a threat to the mental health of anxious individuals [17,18].

Experiencing the unknown due to a perceived lack of information leads to uncertainty, which undermines an individual’s ability to prepare effectively and efficiently for the future, thereby increasing the vulnerability of anxiety-related symptoms [14,16,17,18]. Job-seeking anxiety perceived by college students is caused by job competition, as well as the increase in the period of job preparation. According to several studies, many students spend plenty of time searching jobs on job search portals and the internet, and also seeking consultancy regarding employment [7,12,13,15,18]. Studies have reported that students from health science courses regard the practical experience as one of the most anxiety-inducing aspects [8,9] because it requires them not only to manage social and personal challenges, but also to deal with additional demands to meet the client needs in the clinical setting in comparison with other courses. While choosing a course, college students should consider not only the means of acquiring social and economic status but also their interests and aptitudes [10]. However, current major studies are becoming less meaningful for studying for job preparation, while preparations for jobs, such as the acquisition of qualifications, proficiency in the English language, language training, personally delivered vocational training or work experience are increasing [12].

Job preparation behavior is a process of job exploration, information collection and a capability to engage in the desired job [13,14,15]. In order to cope with job hunting, affiliated educational institutions help students to obtain information related to jobs. Numerous research topics on job stress can be found in domestic literature [6,10,13,18] and studies on the relationship between job stress and physical health have been mainly conducted in the past [15,17,18]. However, there is insufficient research literature on job-seeking anxiety and job preparation among undergraduate students in different majors and the factors that affect them. In particular, studies targeting high-grade students at four-year colleges and the direct relationship between subcategories of job-seeking anxiety and job preparation activities, are meager. 

Furthermore, the novel coronavirus (COVID-19) pandemic hit local economies hard, and the employment pressure among final year college students across all countries dramatically increased since March 2020 [19]. Studies showed a situation made worse by the COVID-19 pandemic [20,21]. Under the influence of COVID-19, employment has produced a lot of uncertainty, and college graduates are more and more confused and anxious in job hunting and preparation [20,21,22], which has influenced many of them to be constantly looking for employment outside of their course. This preparation behavior would be considered to find a job, but seriously increases anxiety. In addition, this study also takes the pandemic into account when considering job-seeking anxiety and preparation behavior. 

The purpose of this study is to attempt to examine and compare the job-seeking anxiety and job preparation behavior among third- and fourth-grade undergraduate students, as well as discovering the factors that affect them. We also hypothesized that there was a significant positive correlation between job-seeking anxiety and job preparation behavior among undergraduate students.

## 2. Materials and Methods

### 2.1. Study Design

This study focused on a descriptive cross-sectional design using a self-reported questionnaire to analyze and identify the job-seeking anxiety and job preparation behavior of undergraduate students belonging to health sciences and social sciences.

### 2.2. Setting and Sample

The study participants were selected from the K’ university, G city, in Korea. Students from the third and fourth grades were chosen to participate in the study. The required sample size was calculated by using the G power 3.1.9 program, at 0.05 level of significance, 0.05, a power of 0.90, effect size of 0.15 and with 13 independent variables. An average of sample size of 195 and over are allowed as per sampling power. However, our study included 360 students and met the sampling power. 

A convenient sample technique was used by following the ethical approval. University students were invited to participate by a recruitment notice and social network system (SNS) and included the study purpose and methods. Students’ responses were obtained in the form of an expression of interest to participate in this study. A total of 400 undergraduate students were called for data collection. The undergraduate students were classified into the following categories: health science and social science (social welfare and education). The survey was conducted by a self-reported questionnaire, and a total of 400 copies were distributed. The final analysis included 360 (90%) surveys, while the remaining 40 (10%) were excluded due to missed data, including the questionnaires that were not completed within the given time period.

### 2.3. Instruments

The study tools that were employed included the Korean version of a self-reported questionnaire. The general characteristics of the students were age, sex, grade, perception of job-seeking behavior, source of information (job-related), desired occupation, self-perception of job preparation and self-perception of job anxiety. Two tools were used to address the job-related anxiety and preparation behavior: (1) 28 items of job-seeking anxiety; (2) 40 items of job preparation behavior. 

The job-seeking anxiety tool was developed by Cho [23]. It consists of 28 items and subscales, including psychological and physical anxiety (10 items), induction of job anxiety (11 items) and cause job anxiety (7 items), measured on a 5-point Likert scale ranging from 1 (strongly not agree) to 5 (strongly agree). The higher scores indicate a higher level of job-seeking anxiety. The reliability of the tool is Cronbach’s α = 0.95. For this study, the Cronbach’s α was 0.96, implying that the study was reliable. 

The job preparation behavior tool was developed by Oh [24], Korea Job Information Service. The tool consists of 40 items and sub-domains, which include formal information of job search (8 items), informal job information of job searches (10 items), preliminary job preparation (15 items) and job preparation behavior (7 items). The scores are measured on a 4-point Likert scale, and the higher scores indicate an extending level of job preparation. The reliability of the instrument in this study is Cronbach’s α =0.96, while in our study, Cronbach’s α = 0.92. 

### 2.4. Data Collection

The researcher randomly selected the students who qualified to participate in this study. Students were invited to participate in the study by an indirect notification due to the COVID-19 outbreak. The selected questionnaire was distributed and collected as per the student’s convenience regarding place and time. Data were collected from November 2020 to February 2021. All of the participants completed written consent prior to filling the self-reported questionnaire, and the total process took about 40 min to complete. All of the collected questionnaires were reviewed as soon as the questionnaire was completed, and the participants were requested to provide supplementary information, if necessary.

### 2.5. Ethical Considerations

In order to perform this study, ethical approval was obtained from the Institutional Bioethics Committee (IRB-320) of K’ University. The purpose and research process were explained, and permission was obtained from each department head prior to the data collection. The participants were informed of the voluntary participation, and they had the liberty to withdraw at any time. Moreover, they were provided with a written consent form, after receiving instruction, regarding the research purpose, methodology, the expected effects and the response. 

### 2.6. Data Analysis 

The collected data were analyzed using IBM SPSS 25.0 program. The socioeconomic characteristics of the undergraduate students were indicated in frequency, percentage, means and standard deviations, respectively. The level of job anxiety and preparation behavior was calculated by independent t-test, ANOVA and Scheffe’s test with a 5% level of significance. Variance inflation factor (VIF) was used to identify the degree of multicollinearity. The relationship between the variables was analyzed by Pearson’s correlation coefficient. Stepwise regression analysis was performed by using the association between undergraduate students’ demographic variables, job anxiety and job preparation behavior. The statistical significance was calculated at the level of *p* < 0.5.

## 3. Results

### 3.1. Comparison of General Characteristics of Subjects by Major Series

The study population included 360 undergraduate students. The general characteristics are shown in Table 1. It can be seen that most of the participants were female (70.8%) and there was a significant difference between male and female students (<0.001). The age group of 22–24 years (55.2%; 52.7%) was the most common in both fields. Most of the students were in fourth grade (62%; 59.1%), and their parents’ monthly income was <200 (10.000) won (44%; 60%). Both the groups demonstrated job-seeking behavior (Yes = 62.4%; 86.4%) and utilized the university job café (56%; 50%) to seek information regarding jobs. The desired job selection for students of health science was professional, related to the same field (78%), while for social sciences students, it was business and others (54.5%). Job-seeking anxiety and preparation were commonly found among both groups. All of the general characteristics showed significant differences at *p* < 0.05 level except preliminary preparation of a job (Table 1). 

### 3.2. Comparison of Job Preparation Behavior and Job-Seeking Anxiety of Undergraduate Students

The comparison of job-seeking anxiety and job preparation behavior is shown in Table 2. The mean total scores of health science students on job-seeking anxiety (F = 6.51 ± 0.76, *p* < 0.001) showed significant differences, and the mean scores of subscales included employment anxiety state (F = 5.81; *p* < 0.001), employment anxiety induction situation (8.91; *p* < 0.001) and employment anxiety induction causes (F = 3.25; *p* < 0.05). 

The total mean scores on job preparation behavior (F = 8.21; *p* < 0.05) showed a higher proportion than job-seeking anxiety. The subscale results of job preparation behavior (F = 8.21; *p* < 0.05) showed a higher association than job-seeking anxiety. The subscales included official search for employment information (F = 8.21; *p* < 0.05), informal search for employment (F = 8.21; *p* < 0.05), information (F = 8.21; *p* < 0.05), preliminary employment preparation behavior (F = 8.21; *p* < 0.05) and authentic employment preparation behavior (F = 8.21; *p* < 0.05). 

The overall findings revealed that job-seeking health science students had the highest job-seeking anxiety (4.45 ± 0.81) and job preparation behavior (4.28 ± 0.76) compared to the social science students, showing a significant difference at *p* < 0.05 level (Table 2).

### 3.3. Correlation between Job-Seeking Anxiety and Job Preparation Behavior of Undergraduate Students 

As a result of the correlation between job-seeking anxiety and job preparation behavior among undergraduate students, a statistically significant correlation was seen between job-seeking anxiety and variables (Table 3) of health science students. In the job-anxiety state, they showed a positive correlation with employment anxiety induction situation (r = 0.32, *p* < 0.01) and employment anxiety induction causes (r = 0.27, *p* < 0.01). A social correlation of science students’ employment anxiety state showed a positive correlation with employment anxiety induction situation (r = 0.24, *p* < 0.01) and employment anxiety induction causes (r = 0.23, *p* < 0.01) (Table 3).

In the findings of the job preparation behavior of health science students, it can be seen that official search for employment information, informal search for employment information (r = 0.18, *p* < 0.001), preliminary employment preparation behavior (r = 0.271 *p* < 0.01) and authentic employment preparation behavior (r = 0.12, *p* < 0.01) showed positive correlations. However, for social science students, employment information had a negative correlation with informal search for employment information (r = −0.29; *p* = 0.047) and official search for authentic employment preparation behavior (r = −0.23; *p* = 0.052) (Table 3). 

### 3.4. Factors Affecting Job-Seeking Anxiety and Job Preparation Behavior of Undergraduate Students

Multiple regression analysis findings of the health science students, scored as a dependent variable, showed that job-seeking anxiety (β = 0.39, *p* < 0.001) was most related, followed by job preparation behavior (β = 0.32, *p* < 0.001). In particular, an explanatory power of these variables was 21.5% (R2 = 0.215, adjusted R2 = 0.205, F = 12.8, *p* < 0.001) in the health science students (Table 4). 

In terms of social science students’ scores, with selected dependent variables, it was revealed that job preparation behavior (β = 0.51, *p* < 0.001) was more relatable than job-seeking anxiety (β = 0.18, *p* < 0.001). An explanatory power of these variables was 39.2% (R2 = 0.392, adjusted R2 = 0.37,4 F = 30.12, *p* < 0.001) for the health science students (Table 4). 

## 4. Discussion

The purpose of this study was to identify the job-seeking anxiety and job preparation behaviors among undergraduate students. Most of the study participants were female in both groups. The maximum age group was 22–24 years, and most of them were fourth grade students. The findings from this study are consistent with those from other studies [6,7,8,9,10,11]. It could be seen that the main causes in female participants are that most of the job-seeking anxiety is due to unknown fear about lack of employment support, followed by the lack of self-confidence, worry, and prospects. These results are consistent with those of most studies conducted on health science students. Job-related anxiety is not a particular phenomenon for health or humanity science students. Actually, it is very common in Korea.

Economic conditions, including a parental monthly income of less than 200 won (Korean Own), were found to be higher in both groups and preliminary preparation of job was also more significant among health science students than social science. This difference is due to the unique characteristics of the students based on their desired job or to enter a standardized job position. It has been reported that preliminary preparation behavior changes with gender and age, and the desired position has a positive effect on the job-seeking behavior (12–14). 

There were significantly more differences in desired job search and job preparation behavior than preliminary job preparation. It was found that the health science students prepared in advance for their desired job and institution, whereas the social science students did not. This was reported based on the employment rate by the department of the Ministry of Education [16]. In addition, these results showed that students feel confident about finding work, but anxious about starting their career [21]. This anxiety has been there since before the current pandemic for many students, but for almost a third, the current circumstances have exacerbated these conditions. Universities need to provide as much support as they can for students who are entering the labor market in such uncertain times and employers need to be mindful of these results in their hiring processes. 

In other words, college students who cannot tolerate uncertainty will have an anxiety tendency when selecting a career because they cannot hope effectively without knowing the correct result [25]. This also showed that anxiety was a common emotional experience in the face of some situations [26]. Since the desired job of the health science department related job is already known well. Additionally, this study showed that the source of information related to the desired job of the students is highly significant. A study related to the use of the university job cafe and job information site [15] showed a significant number of students seeking jobs. This result is similar to the results of studies conducted in Korea [12,13,14,24,25,26,27,28,29]. Therefore, it is necessary for university students who are preparing for a job to freely use facilities and systems that can provide job-related information smoothly through the Internet and provide personalized job information according to their major.

On the other hand, the college curriculum and job-related information on campus can help prepare students for their jobs. These results show that the current universities are reflecting the demands of the industry, future social change, and industrial demand in the university education in order to nurture field-oriented manpower according to government policy [25]. The most preferred occupational fields are education service industry, healthcare, social science, health and social welfare business and personal service industry. 

The results of this study are as follows. First, the degree of job anxiety among the subjects of the study group was higher among health science students than social science students. Numerous studies have reported that students from health science courses regard the limited or interrupted clinical practicum experience as the most anxiety-inducing aspect [8,9]. However, in studies conducted by Choi [5] and Lee [6], it was reported that among four-year college students, the subjects’ job anxiety was 2.66, which was below average. In the subareas of job insecurity, job anxiety was the highest in the health care department. In recent years, youth unemployment has become a serious social problem not only in Korea, but also around the globe [8,9,10,19,20,21,22,30]. In addition, the causes of job anxiety are students’ own low status, low English proficiency and lack of preparation for a job [4,5,9,10,11,14]. Additionally, priority factors for finding a job and received employment training were inversely associated with employment anxiety in this study. Health science students have their priority factors when finding jobs, such as salary, workplace location, etc. However, the ideal employment was in conflict with serious employment situation, which could lead to anxiety [26,27,28]. Therefore, the job-anxiety problem is not just an individual problem. In order to solve the job problem, it is necessary to make efforts at the school and the national level and prepare a customized job preparation program. 

Assessing job preparation behavior, the health science and social sciences were highly different from each other, except for informal search for employment information. There are limited precedent studies on job preparation behaviors for undergraduate students [18,19,22]. However, the development and validation study of the college students’ job preparation test used the Educational Statistics Survey of the Ministry of Education, Science and Technology [16]. These findings are consistent with the job preparation behavior and its subscales; it was highest in all categories of job search, official job search information, unofficial job search information, preliminary job preparation behavior and full-scale job preparation behavior. The study social science group had the highest level of job insecurity compared to the health science. The higher the uncertainty about the job, the higher the preparedness to work.

In college life, mental health is considered as one of the most important aspects to perform a successful social role independently [9,28,29,30]. However, anxiety arising from the preparation process leads to psychological and social difficulties, such as excessive job competition, fear, and depression, as explained in previous studies [5,6,11]. Failure to cope with these issues can lead to extreme emotional problems, such as suicidal thoughts and alcohol and drug addiction [4,12]. Therefore, it is necessary to prepare a way to prevent and solve the problems caused by jobs by including stress management education, which deals with anxiety for college students’ mental health.

The search for official job information refers to the media or internet search, participation in company briefing and job fair participation, while informal job search information refers to job information obtained from family, relatives, friends, professors, and other people [10,23]. Preliminary preparatory action refers to the acquisition of job skills, such as taking college education courses, acquiring various qualifications, and studying English [13,15,17,18]. Actual preparatory actions include writing an application, visiting a company, taking an interview, etc. [11,24,25]. In this way, there are various factors involved in preparing students to take up jobs. Therefore, programs that increase motivation and self-efficacy and group counseling programs should be developed and applied. In addition, full-fledged job preparation activities should establish a job support center within the university and provide various active and continuous services to students.

The result of this study shows that the degree of job anxiety and job preparation behaviors are higher than the middle level in students studying social sciences and health sciences. Therefore, in order to maintain and manage the mental health of college students who are currently anxious about their future, it is necessary to first help them to grow in a sound society through education for their stress management. In addition, they should be provided with a customized job preparation program for each department to help reduce the unemployment of college students and actively support their jobs.

## 5. Limitations

Apart from the study contributions, there are also a few limitations. This study represents most of the undergraduate students; however, some students of three-year majors or interns were excluded. These students should be considered for future studies to get consistent results. The convenience sampling method of study participants posed as another limitation. Therefore, this sampling may not provide an exact outcome or represent the results of the entire population. In addition, self-reported data collection is described as a limitation because it may be exaggerated. More studies using mixed qualitative and quantitative methods are needed for a better understanding of job-seeking anxiety and job preparation behavior of undergraduate students. Furthermore, the anxiety level was higher among health science students due to a lack of confidence in simulation practice and interrupted clinical practicum during the COVID-19 pandemic. These environmental factors have made them more anxious about their procedure-based national license exam and job interviews in a clinical setting.

## 6. Conclusions and Suggestions

This study attempted to examine and compare the job-seeking anxiety and job preparation behavior of undergraduate students. In addition, this study helped by providing insight towards reducing unwanted job-related mental health problems in undergraduate students by comparing and analyzing the job anxiety and preparation behaviors. Based on the above conclusions, we propose the following: First, there are insufficient studies on job anxiety and job preparation behaviors based on different majors; therefore, more studies in this area need to be conducted. Second, it is necessary to develop intervention strategies for employment-related seminars, job search demonstrations and psychological support services to reduce job-seeking anxiety. In addition, it could be explained that the students’ job search should be more personalized by areas to respond to the different profiles. Therefore, this study suggests developing various customized job search programs for undergraduate students that can encourage their career plans and prepare them for employment in accordance with their desired job preparation stage.

### Implications of the Study

This is the first study to focus on job-seeking anxiety and job preparation behavior among university students in different majors with large samples in Korea. The issues regarding job-seeking anxiety and preparation behavior have the most important concern and cause mental health problems including stress, depression, and suicidal ideation, as reported in previous studies [17,18,28,29,30]. This study suggests that the students’ academic or external affairs department must create an effective management strategy to provide mental health promotion and prevention by developing an intervention program, such as a counseling center and job finding guidance for undergraduates during their final year course.

The recommended policy implications have been divided into school health-based counseling and opportunistic job finding based on evidence of existing research. This includes counselling, e.g., psychosocial and mental wellbeing, opportunistic job search programs differing in terms of job findings, the duration between job preparation, and the specific job type based on individual decisions or recommended by professionals or health care counselors.

These policies should not only be implemented in settings in the university; the quality of teaching and mentoring should be emphasized to promote self-confidence in each student’s major area or interest. In addition, nested qualitative research by focus group interviews would be recommended to identify the potential barriers and facilitators to improve intervention and adherence by helping to identify the impact factors and reduce the job-related findings and behavioral changes that intend to help undergraduate job seekers.

## Figures and Tables

**Table 1:** Sociodemographic characteristics of undergraduate students (N = 360).

Characteristics	Categories	Health Science		Humanity and Social Science		(*p*) *
		N	%	N	%	
	Total	250	69	110	31	
Gender	Male	73	29.2	46	41.8	<0.001
	Female	177	70.8	64	58.2	
Age (years)	19–21	72	28.8	37	33.6	<0.001
	22–24	138	55.2	58	52.7	
	≥25	40	16.0	15	13.6	
Grade	Third	95	38.0	45	40.9	0.021
	Fourth	155	62.0	65	59.1	
Parent monthly income (10,000 won)	<200	110	44.0	66	60.0	
	200–400	92	36.8	25	22.7	0.033
	≥400	48	19.2	19	17.3	
Preliminary preparation of job	Yes	188	75.2	75	68.2	0.421
	No	62	24.8	35	31.8	
Source of information	University job cafe	140	56.0	55	50.0	0.008
	Internet and SNS	85	34.0	40	36.4	
	Consultant and others	25	10.0	15	13.6	
Desired occupation	Profession oriented	195	78.0	35	31.8	
	Education and research	40	16.0	15	13.6	0.002
	Business and others	15	6.0	60	54.5	
Job preparation behavior	Yes	204	81.6	87	79.1	0.006
	No	46	18.4	23	20.9	
Job-seeking anxiety	Yes	185	74.0	75	68.2	<0.001
	No	65	26.0	35	31.8	

* = Chi-square test; SNS = Social network service.

**Table 2:** Comparison levels of job-seeking anxiety and job preparation behavior characteristics of undergraduate students (N = 360).

Characteristics		Health Science ^a^	Humanity and Social Science ^b^	t or F (*p*) Scheffé
		(*n* = 250, 69%)	(*n* = 110, 31%)	
		M ± SD	M ± SD	
Job-seeking anxiety	Total	4.45 ± 0.81	3.73 ± 0.55	6.51(<0.001) a > b
	EAS	4.31 ± 0.90	3.26 ± 0.51	5.81 (<0.001)
	EAIS	4.42 ± 0.81	4.31 ± 0.31	8.91 (<0.001)
	EAIC	4.62 ± 0.75	3.62 ± 0.81	3.25 (0.002)
Job preparation behavior	Total	4.28 ± 0.76	4.06 ± 0.81	8.21 (0.001) a > b
	OSEI	4.41 ± 0.83	3.81 ± 0.87	9.72 (<0.001)
	ISEI	3.98 ± 0.78	4.02 ± 0.65	5.24 (0.003)
	PEPB	4.34 ± 0.59	4.49 ± 0.72	5.12 (0.008)
	AEPB	4.39 ± 0.88	3.91 ± 0.94	4.07 (0.014)

M = mean; SD = standard deviation; EAS = employment anxiety state; EAIS = employment anxiety induction situation; EAIC = employment anxiety induction causes; OSEI = official search for employment information; ISEI = informally search for employment information; PEPB = preliminary employment preparation behavior; AEPB = authentic employment preparation behavior.

**Table 3:** Correlations between job-seeking anxiety and job-preparation behavior of undergraduate students (N = 360).

Variables		Categories	1	2	3	
Job-seeking anxiety	Health science	EAS	1			
		EAIS	0.32 *	1		
		EAIC	0.27 *	0.28 **	1	
	Humanity and social science	EAS	1			
		EAIS	0.24 **	1		
		EAIC	0.23 *	0.25 *	1	
**Variables**		**Categories**	**1**	**2**	**3**	**4**
Job preparation behavior	Health science	OSEI	1			
		ISEI	0.18 **	1		
		PEPB	0.21 *	0.34 *	1	
		AEPB	0.12 *	0.37 *	0.41	1
	Humanity and social science	OSEI	1			
		ISEI	−0.29	1		
		PEPB	0.24 *	0.3 *	1	
		AEPB	−0.23	0.29 *	0.19	1

* = *p* < 0.01; ** = *p* < 0.001; EAS = employment anxiety state; EAIS = employment anxiety induction situation; EAIC = employment anxiety induction causes; OSEI = official search for employment information; ISEI = informally search for employment information; PEPB = preliminary employment preparation behavior; AEPB = authentic employment preparation behavior.

**Table 4:** Multiple regression analysis of undergraduate student’s job-seeking anxiety and job preparation behavior characteristics (N = 360).

Variables	B	SE	β	t	*p*	VIF
	Health Science					
Age ^†^	0.11	0.02	0.13	3.72	0.008	1.06
Grade ^†^	0.14	0.03	0.25	3.56	0.004	1.08
Job preparation behavior	0.24	0.04	0.32	3.88	<0.001	1.13
Job-seeking anxiety	0.28	0.04	0.39	5.07	<0.001	1.22
	R^2^ = 0.215, adjusted R^2^ = 0.205, F = 12.8, *p* < 0.001					
	Humanity and Social science					
Age ^†^	0.14	0.12	0.31	5.11	0.003	1.17
Grade ^†^	0.38	0.05	0.44	3.56	0.025	2.64
Job preparation behavior	0.32	0.09	0.51	5.40	<0.001	4.03
Job-seeking anxiety	0.16	0.08	0.18	2.11	<0.001	3.51
	R^2^ = 0.392, adjusted R^2^ = 0.37, 4 F = 30.12, *p* < 0.001					

B = unstandardized regression coefficient; SE = standard error; β= standardized coefficients; VIF = variance inflation factor; ^†^ dummy variable.

## Data Availability

The raw data supporting the conclusions of this article will be made available by the authors, without undue reservation.
